# Diagnosing and Mapping Pulmonary Emphysema on X-Ray Projection Images: Incremental Value of Grating-Based X-Ray Dark-Field Imaging

**DOI:** 10.1371/journal.pone.0059526

**Published:** 2013-03-26

**Authors:** Felix G. Meinel, Felix Schwab, Simone Schleede, Martin Bech, Julia Herzen, Klaus Achterhold, Sigrid Auweter, Fabian Bamberg, Ali Ö. Yildirim, Alexander Bohla, Oliver Eickelberg, Rod Loewen, Martin Gifford, Ronald Ruth, Maximilian F. Reiser, Franz Pfeiffer, Konstantin Nikolaou

**Affiliations:** 1 Institute for Clinical Radiology, Ludwig-Maximilians-University Hospital, Munich, Germany; 2 Department of Physics and Institute of Medical Engineering, Technische Universität München, Garching, Germany; 3 Medical Radiation Physics, Lund University, Lund, Sweden; 4 Comprehensive Pneumology Center, Institute of Lung Biology and Disease, Helmholtz Zentrum Munich, Neuherberg, Germany; 5 Lyncean Technologies Inc., Palo Alto, California, United States of America; 6 SLAC National Accelerator Laboratory, Menlo Park, California, United States of America; 7 Institute of Materials Research, Helmholtz-Zentrum Geesthacht, Geesthacht, Germany; Leiden University Medical Center, The Netherlands

## Abstract

**Purpose:**

To assess whether grating-based X-ray dark-field imaging can increase the sensitivity of X-ray projection images in the diagnosis of pulmonary emphysema and allow for a more accurate assessment of emphysema distribution.

**Materials and Methods:**

Lungs from three mice with pulmonary emphysema and three healthy mice were imaged ex vivo using a laser-driven compact synchrotron X-ray source. Median signal intensities of transmission (T), dark-field (V) and a combined parameter (normalized scatter) were compared between emphysema and control group. To determine the diagnostic value of each parameter in differentiating between healthy and emphysematous lung tissue, a receiver-operating-characteristic (ROC) curve analysis was performed both on a per-pixel and a per-individual basis. Parametric maps of emphysema distribution were generated using transmission, dark-field and normalized scatter signal and correlated with histopathology.

**Results:**

Transmission values relative to water were higher for emphysematous lungs than for control lungs (1.11 vs. 1.06, p<0.001). There was no difference in median dark-field signal intensities between both groups (0.66 vs. 0.66). Median normalized scatter was significantly lower in the emphysematous lungs compared to controls (4.9 vs. 10.8, p<0.001), and was the best parameter for differentiation of healthy vs. emphysematous lung tissue. In a per-pixel analysis, the area under the ROC curve (AUC) for the normalized scatter value was significantly higher than for transmission (0.86 vs. 0.78, p<0.001) and dark-field value (0.86 vs. 0.52, p<0.001) alone. Normalized scatter showed very high sensitivity for a wide range of specificity values (94% sensitivity at 75% specificity). Using the normalized scatter signal to display the regional distribution of emphysema provides color-coded parametric maps, which show the best correlation with histopathology.

**Conclusion:**

In a murine model, the complementary information provided by X-ray transmission and dark-field images adds incremental diagnostic value in detecting pulmonary emphysema and visualizing its regional distribution as compared to conventional X-ray projections.

## Introduction

Chronic obstructive pulmonary disease (COPD) involves progressive airflow obstruction and airway inflammation [1] and represents one of the leading causes of morbidity and mortality throughout the world. [2] Emphysema is a common component of COPD and is characterized by irreversible destruction of alveolar architecture with enlargement of distal airspaces. The diagnosis of pulmonary emphysema as well as its severity assessment largely relies on pulmonary function tests [3]. Spirometry, however, strongly depends on patients’ cooperation and cannot assess the regional distribution of emphysematous changes within the lung. Conventional chest radiography is commonly used to diagnose the presence of emphysema in patients with suspected COPD. Chest radiograms are highly accurate for advanced emphysema [4] but only moderately sensitive in patients with mild to moderate emphysema. [5], [6] Furthermore, assessment of emphysema on chest X-rays shows substantial interobserver disagreement and is unable to quantify the degree of emphysema. [7] Imaging of pulmonary emphysema has been greatly improved with high-resolution computed tomography, however at the cost of exposing the patient to a higher radiation dose as compared to conventional chest radiography. [6] Since emphysema is characterized on CT by abnormally low attenuating lung parenchyma, the presence and degree of pulmonary emphysema can be assessed visually or by densitometry. [8], [9], [10] Although the malignancy risk associated with the radiation exposure from a single CT examination of an adult is – if any – very low, the radiation exposure associated with CT limits its use for frequent follow-up examinations to monitor disease progression in emphysema.

In addition to an accurate diagnosis, assessing the regional distribution of pulmonary emphysema can be crucial for clinical decision-making, e.g., regarding lung volume reduction surgery and endobronchial procedures. [10], [11], [12] Conventional chest X-rays are of limited use in assessing the regional distribution of emphysema. [10] Using CT densitometry, parametric maps of emphysema distribution can be generated by identifying voxels in the lung parenchyma with a CT density below a relative (e.g. 15^th^ percentile) or absolute (e.g. −950 Hounsfield units) threshold. [13], [14] This technique shows good reproducibility [15] and has been validated against pulmonary function tests in clinical trials of pulmonary emphysema. [16], [17] Emphysema distribution can also be assessed by ventilation/perfusion scintigraphy or SPECT, but this offers little additional value over CT scanning [10] and is unable to detect important comorbidities.

Therefore, it would be of great value for emphysema imaging to develop a radiographic projection technology that is more sensitive for mild to moderate emphysema than conventional chest radiography and which would allow for accurate assessment of emphysema distribution on projection images. Such a technology can be expected to facilitate early diagnosis of emphysema, and to frequently obviate the need for CT scanning, thus reducing radiation exposure.

In grating-based imaging, a grating interferometer is introduced into an x-ray projection setup and allows to extract three different contrast modalities [18]: in addition to the transmission signal (equivalent to a conventional x-ray image), grating based x-ray imaging generates a phase-contrast signal as well as a dark-field signal. [18] The phase-contrast signal represents the first derivative of the phase shift [19] while the dark-field signal measures the local small angle scattering of x-rays in the object. [18] Theoretical considerations and experimental data have shown that both phase-contrast and dark-field signals reveal additional information on the specimen, complimentary to the information provided by the transmission signal. [20], [21], [22] In dark-field imaging, the signal strength is determined by small-angle scattering from microstructures on a scale below the spatial resolution of the imaging system [18], [22] thus revealing structural information that is inaccessible for transmission and phase-contrast images. [20] This makes X-ray dark-field imaging a promising technology for lung imaging, since the alveoli that constitute most of the pulmonary parenchyma have a diameter well below the resolution of clinical X-ray projection images.

A recent study demonstrated that diagnosing and mapping pulmonary emphysema is feasible by combining transmission and dark-field signal in grating-based X-ray imaging. [23] Based on the data from this feasibility study, the purpose of the present study was to investigate whether grating-based X-ray dark-field imaging increases the sensitivity of X-ray projection images for pulmonary emphysema and allows for a more accurate assessment of emphysema distribution on projection images as compared to conventional X-ray transmission images alone.

## Materials and Methods

### Ethics and Animal Welfare

This study does not involve human participants or human samples. Animal experiments were performed with permission of the Institutional Animal Care and Use Committee of the district government of Upper Bavaria. Experiments were performed according to national (GV-SOLAS) and international (FELASA) animal welfare guidelines. Mice were kept in fully air-conditioned and pathogen free conditions and had free access to water and rodent laboratory chow at all times. To allow for adaptation, mice were kept in the animal facilities at least 7 days prior to starting the experiments. Mice were visited daily and euthanized if any signs of suffering (such as weight loss >10%, food denial, aggressive or apathetic behavior, heavy breathing, bleeding from mouth or nose) were observed. To ameliorate suffering, mice were anesthetized using intraperitoneal injection of Medetomidine, Midazolam and Fentanyl for both the endotracheal application of elastase and for pulmonary function tests. Following pulmonary function tests, painless euthanasia was performed by cervical dislocation under deep anesthesia.

### Murine Model of Pulmonary Emphysema

Six- to eight-week-old pathogen-free female C57BL/6N mice (Charles River Laboratories, Sulzfeld, Germany) were used throughout this study. To induce pulmonary emphysema, pancreatic elastase was dissolved in sterile phosphate-buffered saline and applied once orotracheally (80 U/kg body weight). Control mice received 80 µl sterile phosphate-buffered saline. [24] Successful induction of emphysema was confirmed by in vivo pulmonary function tests. For pulmonary function tests mice were anesthetized, tracheostomized and connected to a FlexiVent pulmonary function system (Scireq, EMKA Technologies, Paris, France). During the measurement mice were ventilated with an average breathing frequency of 160/min. A snapshot perturbation maneuver was applied to determine the overall dynamic compliance of the respiratory system. Subsequently, forced oscillation technique perturbation maneuvers were conducted to measure tissue elastance. For each parameter, an average of three measurements per mouse was calculated. Mouse lungs were excised 28 days after elastase application, inflated with air and tied up at the trachea.

#### Histopathology

After washing to remove paraformaldehyde, lungs were decalcified in 10% EDTA for 5 days. Subsequently, the specimens were dehydrated and embedded in paraffin. Multiple 10 µm thin sections were prepared in the coronal plane at intervals of 0.5 mm to obtain representative sections covering the entire organ. Sections were deparaffinized, hydrated, stained using a routine Mayer’s hematoxylin and eosin (H&E) staining protocol and dehydrated. Sections were scanned at various magnifications to create digital images.

#### Imaging setup

The Compact Light Source (CLS) is a laboratory-scale synchrotron X-ray source, commercially developed and manufactured by Lyncean Technologies, Inc (Palo Alto, CA, USA). A radio-frequency electron gun and a laser-driven photocathode produce single electron bunches, which are accelerated to an energy level in the range of 20 to 45 MeV in a linear electron accelerator section. The bunch is stored at this energy in a miniature storage ring with a circumference of a few meters. A high-finesse bow-tie enhancement cavity is located at one of the straight sections of the storage ring and is resonantly driven by an infrared laser. At the interaction point the laser and the electron bunch are tightly focused and pass through each other on each revolution of the electron bunch and each cycle of the laser pulse. Through the process of inverse Compton scattering, pulses of X-rays are produced on each revolution. A grating interferometer was placed 16 m from the source. A π/2 phase shift grating with 5.28 µm pitch (design energy 33 keV) and an absorption grating with 5.4 µm pitch were used at the first fractional Talbot distance. The Compact Light Source (CLS) was operated at an X-ray energy of E_peak_ = 36 keV.

#### Image acquisition

Lungs from three mice with pulmonary emphysema and lungs from three healthy mice were placed in formalin filled plastic containers and imaged *ex vivo* in a water bath. For each sample, 11 projections over 180° were acquired by rotating the sample around the tomographic axis. Each dataset consists of a phase-stepping scan of the absorption grating with respect to the phase grating, over one grating period using 16 steps. The exposure time for each phase step was 5 seconds. Median radiation exposure per lung was 34 mGy. [25] All images were recorded using a Varian PaxScan 2520D detector with square pixels of 127 µm x 127 µm and a CsI scintillator.

#### Image post-processing

**Figure 1:**
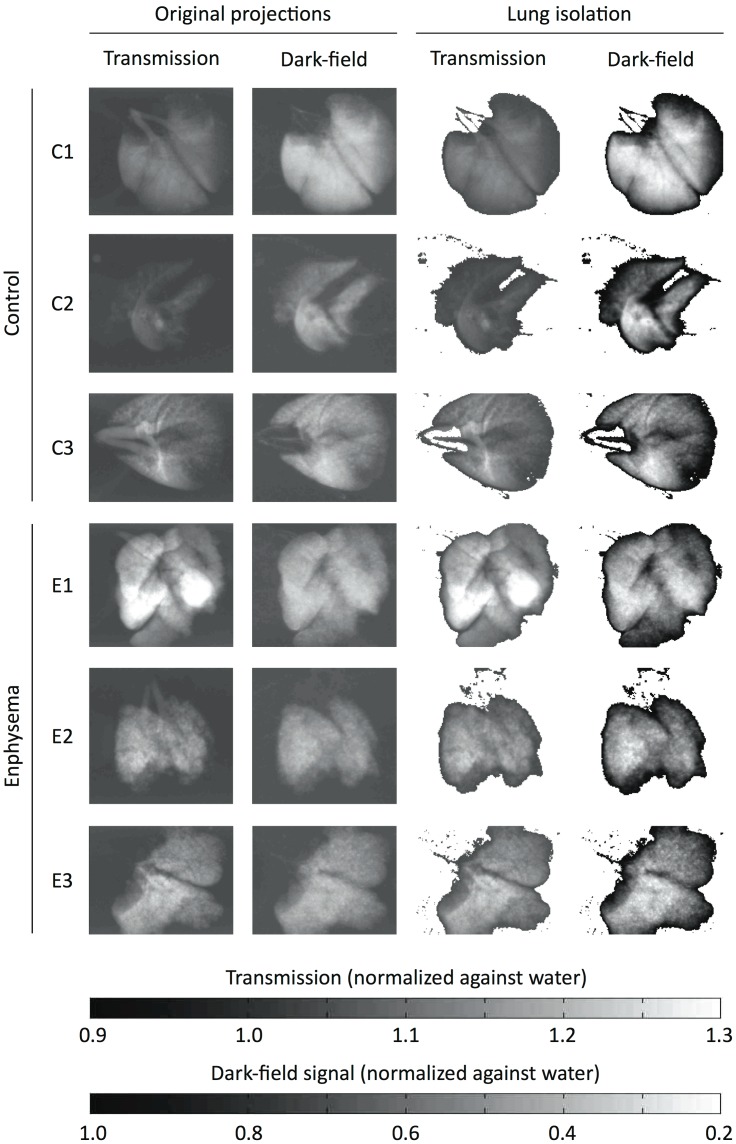
Original and segmented transmission and dark-field projection images. On the left, the transmission and dark-field signal from one original projection image is shown for each control (C1-3) and emphysema (E1-3) mouse. Out of 11 projections acquired for each lung, one sample projection was chosen visually such as to display both lungs with the least possible overlap (thus resembling a frontal chest radiograph). Lung tissue areas were isolated from the images using a dark-field signal threshold for segmentation. The threshold was visually determined as the border of background noise and the signal peak on dark-field signal histograms. This segmentation was used for both dark-field and transmission images, such that identical lung areas were isolated on both images. The resulting isolated images are shown on the right. Note that this segmentation approach validly distinguishes pulmonary parenchyma from background signal and also removes most of the trachea and main bronchi.

Post-processing was performed using software written in-house within Matlab. The effect of the sample on the wavefront was calculated from the raw projections using Fourier analysis, resulting in three different contrast modalities: standard absorption, phase-contrast and dark-field images. Lung tissue areas were isolated from the images using a dark-field signal threshold for segmentation. The threshold was visually determined as the border of background noise and the signal peak on dark-field signal histograms. This segmentation was used for both dark-field and transmission images, such that identical lung areas were isolated on both images (see Figure 1).

#### Data processing and statistical analysis

Data processing and statistical analysis were performed using Microsoft Excel for Mac 2011 (version 14.1.3) and IBM SPSS Statistics for Mac (version 20.0.0.1). Transmission and dark-field signal values normalized against water were recorded for individual pixels as well as median values per individual. From the transmission (T) and dark-field signal (V), we calculated the combined parameter “normalized scatter” defined as S = − (ln V)/(ln T − c). The correction factor c was necessary, since the transmission values were normalized against the water bath outside the specimen container, which does not represent the background of the specimen. The background of the specimen is formed by the formalin within the specimen containers. The transmission signal of the formalin background is slightly higher than the transmission signal of the surrounding water bath, since the x-ray beam passes the plastic container. To correct for this, for each specimen, ROIs were placed in the formalin background inside the containers and in the water bath surrounding the containers. Their values were averaged and the correction factor c was calculated as ln (T_Formalin_/T_Water_). The numerical value of c was 0.02. The resulting parameter S = − (ln V)/(ln T − c) can be regarded as the “normalized scatter” of tissue, i. e. the tissue’s scatter normalized against its transmission. Since both transmission and dark-field signal are logarithmically related to sample thickness, the combined parameter normalized scatter is independent of sample thickness. [23].

Median signal intensities were compared between emphysema and control group using the Mann-Whitney-U-test. To determine the diagnostic value of transmission, dark-field and normalized scatter values in differentiating between healthy and emphysematous lung tissue, a receiver-operating-characteristic (ROC) curve analysis was performed both on a per-pixel and a per-individual basis. Corresponding area under the ROC curves (AUC) were compared using the standard error of the test statistics as derived from the asymptotic variance covariance. [26] In the pixel-based analysis, “optimal” cut-off values for transmission, dark-field and normalized scatter values were determined such as to maximize the respective area under the curve (AUC). To generate parametric maps of emphysema distribution, the difference between pixel values of transmission, dark-field and normalized scatter from the respective “optimal” cutoff value was calculated for each pixel and normalized against the interquartile range of this parameter in the control lungs. The resulting values were color-coded on a scale ranging from 0–1 interquartile ranges. Only deviations towards the “emphysematous” signal characteristic were color-coded such as to identify diseased tissue. The resulting color-coded parametric maps were superimposed onto the transmission images depicted in gray scale and compared to histopathology.

## Results

### Confirmation of Emphysema by *in vivo* Pulmonary Function Tests and Histopathology

Mice in the emphysema group showed increased pulmonary dynamic compliance (median 72.2 vs. 49.8 µL/cm H_2_O) and decreased tissue elastance (median 9.8 vs. 19.9 cm H_2_O/cm). Histopathology showed diffuse enlargement of the distal airways. This confirmed successful induction of emphysema with a phenotype resembling pulmonary emphysema in humans.

### Difference in Transmission, Dark-field and Normalized Scatter Values between Emphysematous and Healthy Lungs

**Figure 2:**
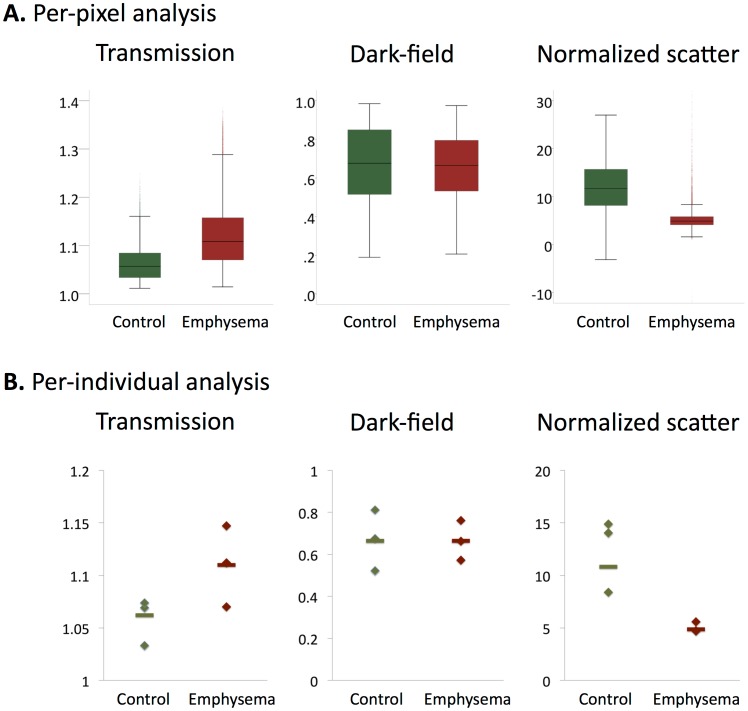
Signal characteristics of emphysematous and control lungs. The distribution of transmission, dark-field and normalized scatter values are shown as boxplots for emphysematous and control lungs in a per-pixel (**A**) and a per-individual (**B**) analysis. For the per-individual analyses median values over all 11 projections of each specimen were used (depicted as rhombs). Additionally, the median value over all projections of all specimens from the respective group is shown (horizontal bar).

In order to evaluate if transmission, dark-field and normalized scatter can identify emphysematous lung tissue, we compared their median signal values between projections from healthy and diseased samples. Normalized scatter was defined as the scatter signal in a given pixel normalized against its transmission signal as described in the Methods section. In a per-pixel analysis, median transmission values relative to water were higher for emphysematous lungs than for control lungs (1.11 vs. 1.06, interquartile range 1.07–1.17 vs. 1.04–1.10, p<0.001, Figure 2A). There was no difference in median dark-field signal intensities relative to water between both groups (0.66 vs. 0.66, interquartile range 0.52–0.81 vs. 0.48–0.85, Figure 2A). Median normalized scatter was significantly lower in the emphysematous lungs compared to controls (4.9 vs. 10.8, interquartile range 4.1–5.8 vs. 7.5–14.7), p<0.001, Figure 2A). Similar results were obtained in a per-individual analysis (Figure 2B). This data suggests that while both transmission and normalized scatter, but not dark-field signal, differ between healthy and affected samples, the normalized scatter value allows the clearest differentiation between the two.

### Diagnostic Value of Transmission, Dark-field and Normalized Scatter Values for the Detection of Pulmonary Emphysema

**Figure 3:**
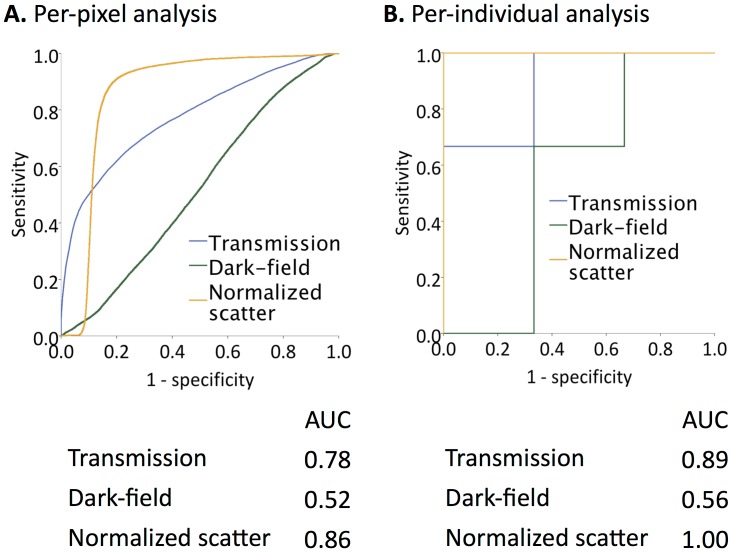
ROC Analysis. Receiver-operator-characteristic (ROC) curves are shown both in a per-pixel (**A**) and a per-individual (**B**) analysis. For the per-individual analyses median values over all 11 projections of each specimen were used. The corresponding areas under the curve (AUCs) are listed below.

To compare the diagnostic value of transmission, dark-field and normalized scatter, a receiver-operating-characteristic (ROC) curve analysis was performed both on a per-pixel and a per-individual basis. In a per-pixel ROC analysis for the detection of emphysema, the AUC for the normalized scatter value was significantly higher than for transmission (0.86 vs. 0.78, p<0.001) and dark-field value (0.86 vs. 0.52, p<0.001) alone (Figure 3A). Cut-off values maximizing the AUC for all three signals and their corresponding AUCs are shown in table 1. Again, similar results were obtained in a per-individual analysis (Figure 3B). Interestingly, transmission and normalized scatter ROC curves show very distinct characteristics in the per-pixel analysis (Figure 3A). Normalized scatter showed an extremely high sensitivity for a wide range of specificity values. For example, for a specificity of 75%, the sensitivity of normalized scatter was 94% compared to a sensitivity of 67% for transmission. Reversely, transmission showed higher specificity for emphysematous lung tissue at low sensitivity values. At 25% sensitivity, specificity for normalized scatter was 90% compared to 98% for transmission.

**Table 1:** Cut-off values with maximized AUC in the per-pixel analysis.

	Cut-off	AUC
Transmission	1.087	0.50
Dark-field	0.715	0.27
Normalized scatter	7.00	0.73

### Diagnostic Value of Transmission, Dark-field and Normalized Scatter Values for Assessing the Regional Distribution of Pulmonary Emphysema in Correlation to Histopathology

**Figure 4:**
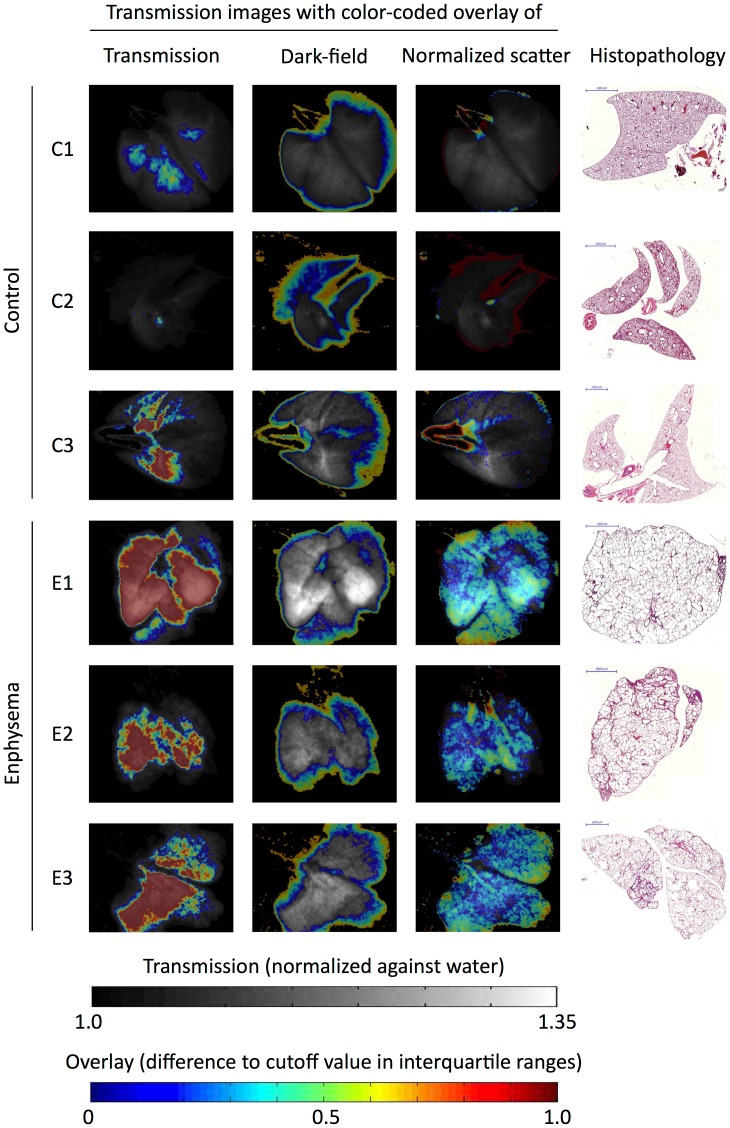
Parametric maps of emphysema distribution. To generate parametric maps of emphysema distribution, the difference between pixel values of transmission (left column), dark-field (second column from left) and normalized scatter (third column from left) from the respective “optimal” cutoff value (determined in the ROC analysis to maximize AUC) was calculated for each pixel and normalized against the interquartile range of this parameter in the control lungs. The resulting values were color-coded on a scale ranging from 0–1 interquartile ranges. Only deviations towards the “emphysematous” signal characteristic were color-coded such as to identify diseased tissue. The resulting color-coded parametric maps were superimposed onto the transmission images depicted in gray scale. Histopathologic images obtained by Mayer’s hematoxylin and eosin (H&E) staining of the corresponding specimen are shown (right column).

Histopathology showed marked emphysema distributed homogeneously throughout all three lungs in the emphysema group and preserved healthy lung architecture in all lungs in the control group (Figure 4). To determine if this disease distribution can be accurately predicted based on our imaging data, we generated parametric maps of emphysema distribution based on the deviation of each pixel values from their respective “optimal” cutoff value (determined in the ROC analysis to maximize AUC). When using transmission values to depict the regional distribution of emphysema, there are obvious differences between healthy and emphysematous lung tissue. However, since the thicker central portions of the lung will inevitably result in higher transmission than thinner peripheral portions, central regions of control lungs C1 and C3 were erroneously displayed as diseased while large peripheral portions of all emphysematous lungs are erroneously displayed as healthy (Figure 4). Using the dark-field signal alone was clearly inappropriate to depict the regional distribution of emphysema. Instead, the peripheral portions of all lungs are identified as diseased (Figure 4). Using the normalized scatter signal to display the regional distribution of emphysema yields color-coded parametric maps, which clearly show the best correlation with histopathology. All three emphysematous lungs can be recognized as being homogeneously diseased. In the control lungs, only the large airways and a minimal rim of peripheral lung tissue are falsely displayed as emphysematous (Figure 4).

## Discussion

A recent proof-of-concept study has demonstrated that it is feasible to differentiate emphysematous from healthy lung tissue by combining transmission and dark-field signals from grating-based X-ray projection images. [23] Based on this data, we show in this manuscript that this approach is superior to X-ray transmission images alone in the diagnosis of pulmonary emphysema and in assessing its regional distribution. Specifically, we demonstrate in the ROC analysis for detecting emphysema that the AUC of normalized scatter (which we defined as local scatter normalized against transmission relative to water) is significantly higher than the AUC of transmission or dark-field signal alone. We further showed that the normalized scatter can be used to generate color-coded parametric maps of emphysema distribution that show a better correlation with histopathology than can be achieved using transmission or dark-field signal alone. We found that the ROC curves of normalized scatter and transmission show a distinct pattern in the per-pixel analysis. Normalized scatter shows an extremely high sensitivity for pulmonary emphysema at intermediate specificity.

On cross-sectional CT images, the quantitative assessment of transmission values for each voxel known as densitometry allows valid assessment of emphysema distribution. [10] Densitometric mapping of pulmonary emphysema is not feasible on radiographic projection images, since the transmission value of each pixel will not only reflect parenchymal density but largely depend on the local thickness of lung parenchyma. This is reflected in the partly inadequate maps of emphysema distribution that our data generates based on transmission values alone. Defining normalized scatter as the projected small angle scattering normalized against transmission renders this parameter largely independent of sample thickness thus allowing valid mapping of emphysema on projection images.

The results of this study have to be seen in the context of the study design. The X-ray source used in this study was a laser-driven compact X-ray source also referred to as a laboratory-scale synchrotron. This X-ray source produces a beam with many of the characteristics of synchrotron radiation, such as near-monochromaticity. In contrast to synchrotron facilities which can be up to several 100 m in diameter, the Compact Light Source used in this study measures about 5×2 m. Current routine medical imaging, in contrast, uses conventional X-ray sources with polychromatic spectra. Unlike analyzer-based and standard interferometric methods, grating-based X-ray imaging has relatively low requirements on spatial or temporal coherence and can therefore be readily performed with conventional X-ray sources. [19], [27] This has been demonstrated in phantom models [21], animal [28] and human [29] tissue samples. Nevertheless, future studies are needed to confirm our data regarding the diagnostic value of grating based X-ray imaging for pulmonary emphysema using a conventional X-ray source.

Our study was performed using *ex vivo* mouse lungs. For future applications of grating-based X-ray lung imaging, overlying structures of the thorax, particularly ribs and fur, as well as breathing artifacts may confound both transmission and dark-field signals. These challenges have to be addressed in subsequent studies. In order to avoid breathing artifacts, the exposure time of 5 s used in this study appears short enough to stop ventilation in anesthetized and mechanically ventilated rodents.

The median radiation dose of our setup was 34 mGy. [25] This is considerably higher than the radiation dose associated with a standard clinical chest x-ray. [30] However, as demonstrated previously, reducing the number of phase steps and the spatial resolution can reduce this dose to approximately 2 mGy without significant information loss. [25] Also, the setup used in this study is not designed for *in vivo* experiments and has therefore not been optimized for dose efficiency. Technical optimization of the setup is likely to result in further decreases in radiation dose approaching that of a conventional radiograph.

CT and CT densitometry are the current clinical standard for diagnosing and assessing the regional distribution of pulmonary emphysema. The novel aspect of our study is the incremental value for combined transmission and dark-field imaging for plain radiographs. Our study did not compare this approach to CT or CT densitometry. Grating-based imaging allows to acquire not only plain radiographs but also tomographies which can again be reconstructed as transmission and dark-field images. Future studies are needed to assess whether there is also an incremental value for combined transmission and dark-field CT over conventional transmission CT alone.

It is known that the results of CT lung densitometry are influenced by lung volume, which is why densitometric results should be corrected for lung volume. [31] Since we did not perform measurements at different lung volumes, we cannot assess whether the normalized scatter (dark-field signal normalized over transmission signal) is also influenced by lung volume. This issue needs to be addressed in subsequent studies.

### Conclusions

Grating-based X-ray imaging allows the acquisition of projection images from which both transmission and dark-field signals can be extracted. The complementary information provided by X-ray transmission and dark-field images adds incremental diagnostic value in detecting pulmonary emphysema and visualizing its regional distribution as compared to conventional X-ray projections. In the future, this may allow to detect human pulmonary emphysema more accurately on chest X-rays and assess its regional distribution without the use of CT.
